# Impact evaluation of a dementia-friendly community mural: Planting a seed for change

**DOI:** 10.1177/14713012251333864

**Published:** 2025-04-11

**Authors:** Laura Garcia Diaz, Evelyne Durocher, Paula Gardner, Carrie McAiney, Lori Letts

**Affiliations:** School of Rehabilitation Science, Faculty of Health Sciences, Institute for Applied Health Sciences Building, 3710 McMaster University, Hamilton, ON, Canada; Department of Communication Studies and Media Arts, Faculty of Humanities, 3710 McMaster University, Hamilton, ON, Canada; School of Public Health Sciences, 8430 University of Waterloo, Waterloo, ON, Canada; Schlegel-UW Research Institute for Aging, 8430 University of Waterloo, Waterloo, ON, Canada; School of Rehabilitation Science, Faculty of Health Sciences, Institute for Applied Health Sciences Building, 3710 McMaster University, Hamilton, ON, Canada

**Keywords:** community mural, dementia, dementia-friendly communities, public art, stigma

## Abstract

Stigma against persons living with dementia negatively impacts their quality of life. One of the aims of dementia-friendly community initiatives is to reconstruct public perceptions of dementia through dementia awareness campaigns. In this paper we present the findings of an evaluation of the impact of a Canadian dementia-friendly community mural on raising awareness about dementia, and lessons learned from the process undertaken to create the mural. Using a practical participatory evaluation research approach, the research questions and data collection methods were co-created with the team that led the community mural, which was comprised of four people living with dementia. Numerous data collection methods were used to support this outcome evaluation including observations, interviews and focus groups, and social media tracking. Findings indicate that while the process of creating the mural, and the mural itself, have planted a seed for increased dementia awareness and inclusion of persons living with dementia in the community, to create social change complementary awareness raising efforts are needed.

## Introduction

Dementia is a general term that describes a group of symptoms caused by disorders affecting the brain (World Health Organization [WHO], 2012). The disorders are progressive in nature and may lead to changes in thinking, memory, emotional regulation, judgement, and language (WHO, 2021a). Dementia is one of the major causes of dependence and disability worldwide (Frankish & Horton, 2017) and in 2012 it was recognized as a global health priority (WHO 2012).

Stigma against persons living with dementia negatively impacts their quality of life (Nguyen & Li, 2020). As a result of public misunderstanding and lack of knowledge about dementia, many persons living with dementia have shared feeling excluded from their social circles and no longer being taken seriously by their friends and family (van Wijngaarden et al., 2019). Additionally, negative societal attitudes and beliefs about dementia can create fear of a dementia diagnosis and how a person and their caregivers may be treated after a diagnosis, which can lead to delays in help-seeking behaviors (Parker et al., 2020). Relatedly, common stereotypes of persons living with dementia include lower cognitive competence and the person being aggressive, which can result in discrimination towards them by healthcare providers (Gove et al., 2016; Nguyen & Li, 2020). The negative impact that stigma can have on the lives of persons living with dementia and their caregivers highlights a need for interventions and campaigns aimed at reconstructing public perceptions of dementia.

Dementia-friendly community (DFC) initiatives are intended to support the quality of life of persons living with dementia by changing and/or creating social and physical environments to promote equal access to opportunities, public spaces and services, and prevent discrimination (WHO 2021b). An important component of DFC initiatives is raising awareness and public knowledge of dementia (Alzheimer’s Disease International, 2016). Dementia awareness campaigns are typically aimed at achieving one or more of the following: increasing understanding about dementia, including recognition of early signs and symptoms; reducing stigma and discrimination towards persons living with dementia and their care partners; educating the public about risk reduction behaviors; and sharing stories about living with dementia (Matsumoto et al., 2021; Phillipson et al., 2019).

Community murals have been described as a form of public art that promote “community pride and commitment to justice while teaching outsiders about the struggles of traditionally oppressed people” (Conrad, 1995, p. 98). Although community murals have not traditionally been used to raise awareness about dementia, historically, murals have been used as a way to increase knowledge about a local issue, and to encourage further public discourse and community action to address the issue (Burnham, 2011; Szőke & Parizeau, 2019). Community murals have been used to resist harmful public narratives and increase knowledge of a number of topics including suicide (Mohatt et al., 2013), mental health (Ho et al., 2016), COVID-19 (McEwan et al., 2022) and gentrification (Schrader, 2017). However, to our knowledge, community murals have not been used to raise awareness about dementia.

In a discussion paper published over two decades ago, Hall and Robertson (2001) argue that despite claims that public art could create social change, “very little satisfactory evaluation of these claims has taken place” (*p* 18). Twenty years after the discussion paper was published, findings from a systematic review on the impact of public art on cities, places and people’s lives indicate that there continues to be a lack of studies on how communities experience, perceive and engage with public art (Cheung et al., 2021). Without evidence of the impact of murals, it cannot be argued that community murals help to create social change. To advance knowledge about the impact of community murals in creating social change, specifically in communities working towards being dementia-friendly, this study evaluated the impact of a DFC mural aimed at raising awareness about dementia. This impact evaluation sought to answer the following research questions:

 1. Does the mural capture the public’s interest?
2. Does the mural encourage the public to learn more about the work of the team leading the project?
3. Does the mural encourage and inspire the public to act?
4. Does the mural encourage the public to have conversations about dementia and/or DFCs with their social circle?
5. What lessons can be learned from the process undertaken to create this community mural?

## Methods

We first provide an overview of the community mural project, which is followed by a description of the methods used to evaluate the mural’s impact.

### “Dementia-friendly communities for everyone” mural project

In 2020, the Hamilton Council on Aging received funding from the Public Health Agency of Canada to engage persons living with dementia, care partners, and other stakeholders in the development, implementation, and evaluation of DFCs in Hamilton and Haldimand County (located in Ontario, Canada). Haldimand County has a population of approximately 49,000 people, with an estimated 21% of residents being 65 years and older (Statistics Canada, 2021). Haldimand County is composed of six small urban areas: Caledonia, Dunnville, Hagersville, Jarvis, Townsend and Cayuga.

**Figure 1.**
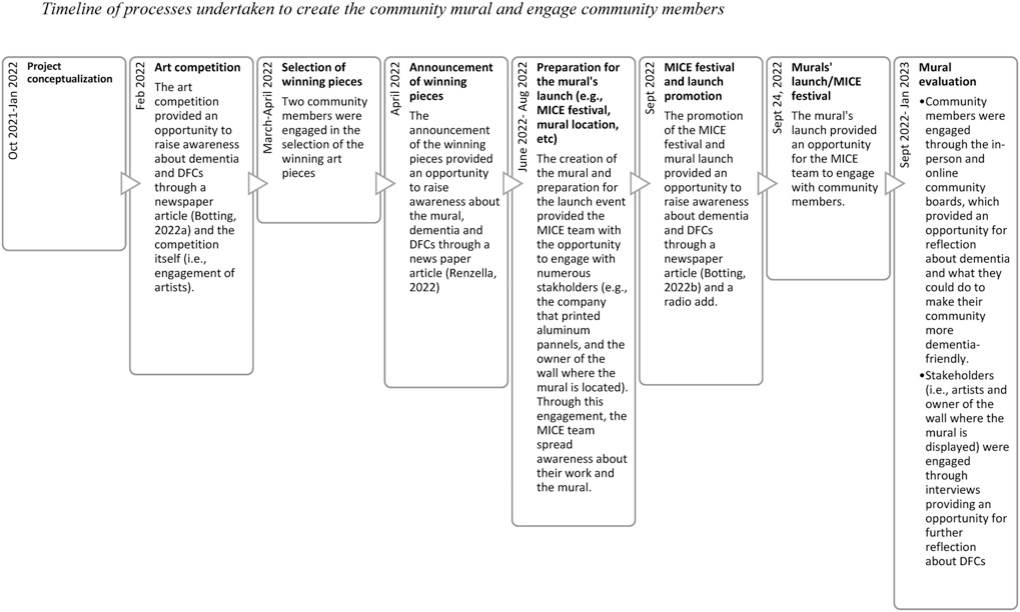
Timeline of processes undertaken to create the community mural and engage community members.

To lead the initiative in Haldimand County, the Memory Inclusive Communities Everywhere (MICE) team, a group of four people living with dementia who reside in Haldimand County, was established in the summer of 2021. For their first project, the MICE team lead a community mural project to achieve their goal of raising community awareness about dementia. The MICE team intentionally chose to create a community mural as their first project because both the process of creating the mural, and the mural itself, presented opportunities to actively engage with community members to raise awareness about dementia and promote inclusivity. Figure 1 provides a timeline of the process undertaken to create the community mural, including how community members were engaged throughout the process.

In February 2022, residents of Haldimand County were invited by the MICE team to submit an original two-dimensional art piece to communicate what “Dementia Friendly Communities for Everyone” might look like (Botting, 2022b). Thirteen artists submitted their artwork and three winning pieces created by Andrea Bridge, Sarah Butcher, and Gina McIntee were selected to be displayed on a community mural (Renzella, 2022). Themes depicted in the winning pieces included messaging about dementia impacting people of all ages and backgrounds, symbols associated with Haldiman county, and imagery to illustrate the strength and hope that community support and inclusion create. The pieces were also intended to encourage community members to learn more about dementia. More information about the artists and their pieces is provided at https://coahamilton.ca/micehaldimand/

The winning pieces were selected by a panel of five judges consisting of members of the MICE team along with invited community members. To support the selection process, the judges were provided with a package that included photos of each of the 13 submissions along with the description provided by the artists. Judges were asked to score the work in six categories (relevance and impact; alignment with the panel’s vision of a DFC; positive and attention grabbing; inclusive design; quality and originality; stimulates imagination) using a scale from 1 (poor) to 5 (excellent). Judges could also give two bonus points if the art reflected the Haldimand county community and was appealing, for a maximum score of 32 points. Judges were asked to score the submissions prior to attending a review meeting during which the final pieces were selected. One of the selected pieces was created by a member of the MICE team; this individual did not participate in the selection of process and re-joined MICE team meetings once the winning pieces had been selected.

**Figure 2.**
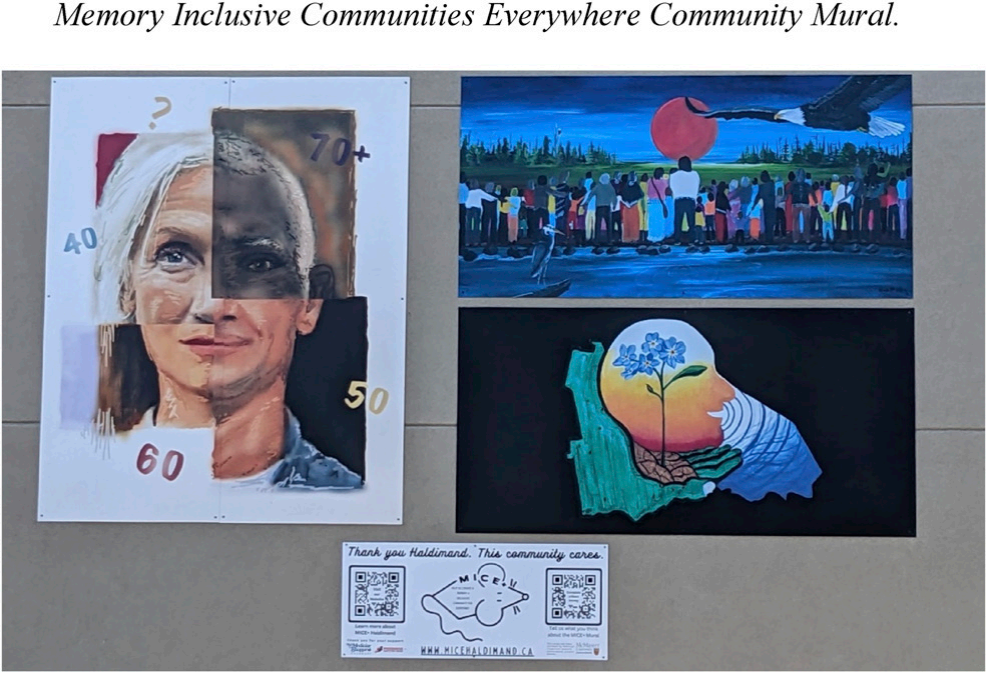
Memory Inclusive Communities Everywhere Community Mural. These art pieces were created by Andrea Bridge (left), Gina McIntee (top, right), and Sarah Butcher (bottom, right).

The three winning art pieces were photographed, enlarged, and adhered to an aluminum panel. The mural was displayed on the outside wall of a pharmacy in Caledonia (see Figure 2 for a picture of the mural). The mural was unveiled in September 2022, during World Alzheimer’s month (Botting, 2022a). For the unveiling of the mural, the MICE team organized a community festival that included a community fair and marketplace, free seminars, children’s crafts, and “memory Olympics”. For the memory Olympics, community members were asked to complete several tasks while undergoing a dementia simulation (e.g., wearing glasses to blur vision). The festival was planned in accordance with an asset-based community development approach (Rahman & Swaffer, 2018), which is grounded in the premise that community members can drive community development processes themselves by identifying community needs and responding to them through the identification and mobilisation of assets. Hence, this project presents an example of an initiative that was designed in relation to “what people and communities already possess and are capable of doing” (Rahman & Swaffer, 2018, p. 132).

### Outcome evaluation methods

A practical participatory evaluation (P-PE; Cousins & Whitmore, 1998) research approach and a mixed method design (Creswell & Plano, 2017) were used for the evaluation. The core premise of a P-PE is that stakeholder engagement in the evaluation process should enhance the relevance, ownership and utilization of evaluation outcomes (Cousins & Whitmore, 1998). With the MICE team’s consent, LGD (principal investigator) attended all MICE team meetings from September 2021- December 2022. To ensure that this outcome evaluation would support the objectives of the MICE team, the MICE team dedicated three team meetings to the evaluation of the mural. LGD facilitated these meetings, and together with the MICE team, the research questions and data collection methods were decided upon. For the other meetings, LGD sat primarily as an observer to gather information about processes undertaken for the project.

### Data collection methods

There is limited guidance about how to evaluate the impact of public art (Milne & Pojani, 2022). Gressel (2012) has proposed analyzing media coverage, conducting observations, and administering surveys as data collection methods that can be used to evaluate the impact of public art. Taking into consideration Gressel’s (2012) propositions, multiple data collection methods were used to support this outcome evaluation (refer to Table 1).

**Table 1.** Research questions and data collection methods.

Evaluation question	Data collection methods
Does the mural capture the public’s interest?	• Observations
	• Interviews and focus group
	• Social media tracking
	• Media request tracking
Does the mural encourage the public to learn more about the work of the MICE team?	• Number of QR code scans (QR code directed people to MICE’s website)
Does the mural encourage and inspire the public to act?	• In-person and online community boards
Does the mural encourage the public to have conversations about dementia and/or dementia friendly communities with their social circle?	• Online community board
	• Interviews
What lessons can be learned from the process undertaken to create this community mural?	• Interviews and focus group
	• Findings from collected data (i.e., all data sources)

#### Community boards

To encourage members of the public to think about what they can do to make their community more dementia-friendly, on the day the mural was launched, LGD set up two community boards by the mural. Each board had one question on it, “How does it feel to have this mural in our community?” and “What will you do to make our community more dementia-friendly?” Cue cards and pens were available for individuals who wished to answer the questions. An “online community board” consisting of an online survey was also developed for individuals who wanted to answer the questions at a later time, and to allow for a longer data-collection period. The survey included the two questions posted on the in-person community boards and a third question to assess whether the mural encouraged the public to have conversations about dementia and/or DFCs with their social circle, “Have you had conversations about the mural with your friends or family?“. Participants were able to select one of three answers, each with a corresponding follow-up question:

 • Yes → Could you please share with us what the conversation was about?
 • No → Is there a reason why you have not had a conversation about the mural with your friends or family?
 • Not yet, but I will be talking about this with my friends or family in the future → Could you please share with us what you plan on talking with your friends or family about?

A QR code, which directed members of the public to the survey, was adhered to the mural at the launch event, and was included in promotional materials. The online survey was available for two months.

#### Observations

To evaluate whether the mural captured the public’s interest, using non-participant observation (Ciesielska & Boström, 2018), LGD observed and tracked the numbers of times someone slowed down to look at the mural, took a picture of/with the mural, appeared to stop to read the mural’s description, and/or appeared to be discussing the mural with the person(s) with whom they were. To perform these observations, LGD sat on a bench placed in front of the mural. Observations occurred the day after the launch of the mural; the weekend following the launch of the mural; and two, four, six and eight weeks after the launch.

#### MICE website tracking

To assess whether the mural encouraged members of the public to learn more about the work of the MICE team, a QR code was developed directing individuals to MICE’s website (https://www.micehaldimand.ca/). The number of people who accessed the hyperlink through the QR code was tracked for two months. The QR code was displayed by the mural and in the launch event’s program.

#### Social media posts and media requests

To evaluate whether the mural captured the interest of the public, for the two months following the launch, the research team tracked the number of individuals who engaged with social media posts posted by community partners (e.g., likes, comments and shares) and media requests to learn more about the mural and the work of the MICE team. LGD used her personal social media accounts to track publicly available social media posts about the mural on Twitter, Instagram and Facebook.

#### Interviews and focus group

To understand what lessons were learned in developing and implementing this project, in December 2022, the MICE team was invited to participate in a focus group with LGD. Additionally, the project coordinator, the artists who won the mural competition, and the pharmacist who provided wall space outside of his pharmacy to display the mural, were invited to participate in an interview with LGD. Focus group and interview participants were asked questions about their perceptions of the impact of the mural and lessons they learned from their involvement with this project. Questions included “Would you do another mural in the future? Please explain” and “Do you think, so far, the mural has achieved what you had hoped?” The focus group and interviews were conducted using the Zoom platform. Only the audio recordings were used for this study. Table 2 provides the evaluation’s timeline.

**Table 2.** Impact evaluation timeline.

Community boards	Observations	MICE’s webpage, social media posts and media requests tracking	Interviews and focus group
Sept 24- public launch: In-person community board	Sept 25–3pm-5pm	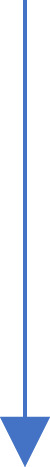	
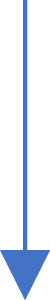	Sept 30–9am-11a.m.		
	Oct 1–10am-12p.m.		
	Oct 8–12pm-2pm		
	Oct 16–11:30am-1:00p.m.		
	Oct 24–10am-12p.m.		
	Nov 24–130pm-230p.m.	Until November 24, 2022	Dec 2022-Jan 2023
Online board (until Nov 24, 2022)			

## Ethics approval

This study received approval from the Hamilton Integrated Research Ethics Board (Project 15031). Informed consent was obtained from all participants. For the community boards, implied consent was obtained by including information about the evaluation on the first page of the online survey and at the top of the community murals, with the statement “if you provide comments on this board/survey, it will be understood that you have consented for your answers to be used to the research project”.

### Data analysis

The qualitative data (community boards, focus group and interview transcripts, and comments on social media posts) and quantitative data (observation counts, social media and media requests tracking, and tracking of engagement with MICE’s webpage) were equally important in addressing the research questions.

LGD and LL analyzed the qualitative data using content analysis (Elo & Kyngäs, 2008). Content analysis is used to examine text intensively for the purpose of classifying text into categories that represent similar meanings (Elo & Kyngäs, 2008). Initial coding for the focus group and interview transcripts was conducted in the Dedoose platform (SocioCultural Research Consultants LLC, 2019). Once initial codes were finalized, all codes and associated quotations were transferred to an excel spreadsheet where they were further refined. The community board responses were imported to an excel spreadsheet. LGD went through the community board responses first and developed a coding sheet which was used by LGD & LL to analyse the data. Once all data from the community boards were organized into codes, codes were grouped into categories. Lastly, the qualitative information from the community boards was quantified by providing a summary of the number of responses that fell under each category.

Quantitative data consisted of: tracking number of times members from the public interacted with the mural; tracking of likes, comments and shares of social media posts posted by community partners; tracking of media requests; and tracking of how many people accessed a hyperlink directing them to MICE’s website via QR code. The data were tracked over time to see if there was an increase in engagement with the mural after media events, social media posts and other communication strategies. Excel was used to generate descriptive statistics for the numeric variables.

Integration of the qualitative and quantitative data happened at the interpretation and reporting stages using a weaving narrative approach (Fetters et al., 2013). This approach involves including both quantitative and qualitative results together (through narrative) on a topic-by-topic basics. LGD shared preliminary findings with the MICE team and discussed with them the meaning and significance of findings. The MICE team’s feedback was integrated in the interpretation of findings.

## Results

For reporting purposes, we first provide a description of participation rates by type of data, followed by the evaluation findings. The results are organized and presented by research question.

### Description of participation rates by type of data

Twenty people contributed to the in-person community boards; 34 contributed to the online survey. LGD conducted seven observations between Sept 25 to Nov 24, totalling 13 hours (refer to Table 2 for the breakdown of hours and times of day). During the two-month period of data collection, there were zero scans of the QR code directing people to MICE’s website. Due to lack of data regarding user engagement with MICE’s website, the research team decided to include website views from August to November as a data source to determine if a pattern could be noticed between views and events associated with the mural (e.g., the launch event).

Over the two-month data collection period, three social media posts by community partners (on Twitter) and two social media posts by members of the public (one in Instagram, one in Facebook) were identified. Both social media posts by members of the public were made by one of the artists (one in Facebook, the other in Instagram). The posts from the community partners had fewer than five likes each and no comments; the Facebook post from the artist had 42 likes and the Instagram post had 12 likes.

Following the launch of the mural, the MICE team had one media request from the local newspaper for more information about the mural and their work. Lastly, five individuals participated in the interviews (project coordinator, pharmacists and the three winning artists) and all members of the MICE team participated in the focus group. The MICE team member who created one of the winning art pieces participated in a focus group and in an interview. To avoid redundancy, she was interviewed after she had participated in the focus group and was asked if she wanted to elaborate on anything else, or if as an artist, there was something related to the process about which she wanted to comment.

### Did the mural capture the public’s interest?

The observation tracking sheet was originally created to capture information about engagement with the mural by individuals who walked by it. However, the mural was located in a heavy vehicular-traffic area with minimal foot-traffic; therefore, the tracking sheet was modified to capture interactions with the mural from people in cars. A traffic light was near the mural allowing for the observation of passengers in cars while they were waiting at the red light. Based on the observations, on average, one person per hour took a picture of the mural while walking by, someone appeared to be looking at the mural from their car 10.5 times per hour, 1.4 people per hour appeared to be having conversations about the mural in their car, and less than one person per hour appeared to be reading the description of the mural while waking by it (refer to Table 3). Notably, we did not record the number of cars that passed by the mural per hour, and therefore, cannot report on whether our findings indicate that a high or low percentage of people appeared to be intrigued by the mural.

**Table 3.** Observation results.

	Number of times that someone took a picture of the mural while walking by it	Number of times that someone appeared to be looking at the mural from their car	Number of people that appeared to be having conversations about the mural in their car*	Number of times that someone appeared to be reading the mural’s description
13 hours of observation	13	137	18	8
Number of people on average per hour	1	10.5	1.4	0.62

*This number was calculated by counting the people in the car that appeared to be having a conversation about the mural. In all cases, two people per car were counted (i.e., 9 cars, for a total of 18 people).

We identified a theme in the analysis of the interviews and focus group noting that *the mural has planted a seed for change*. As described by a focus group participant and an interview participant respectively, “*the mural has started conversations and that’s important*” (07), and “*the semi-permanence of the mural allows for a constant reminder to be more inclusive and kinder*” (01). The permanence of the mural was described by participants as being important for starting to raise community awareness about dementia. Although findings from our observations, interviews and the focus group indicate that the mural has captured the public’s attention, lack of media requests and engagement with MICE’s website and social media posts suggests that the mural has not encouraged members of the public to learn more about the work of the MICE team.

### Did the mural encourage the public to learn more about the work of the MICE team?

**Figure 3.**
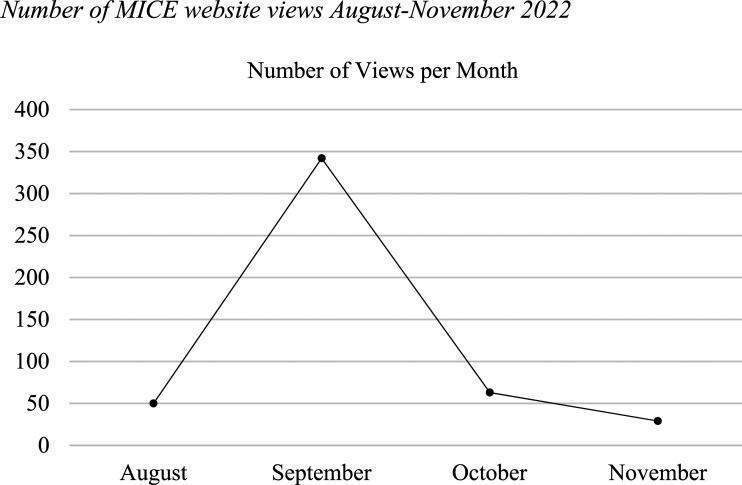
Number of MICE website views August-November 2022.

From reviewing MICE’s website views from August to November, we found that there was a spike in views in September (342 views, compared to 50 views in August, 63 in October and 29 in November), which is the month in which the mural was launched (refer to Figure 3). Review of website views for the month of September indicate that September 23^rd^ (the day before the launch for the mural) had the most views, with 41 users engaging with the website. However, views did not increase after the mural’s launch suggesting that the mural did not encourage the public to visit MICE’s website to learn mote about their work.

### Did the mural encourage and inspire the public to act?

From the 54 community board responses to the question “*What will you do to make our community more dementia-friendly?*” 52 people indicated that they will be doing something to make their community more dementia-friendly, and two responded that they will not be doing anything. Five categories of things that people will be doing were identified (refer to Table 4): support all community members, regardless of their diagnosis (32%); seek more dementia education (30%); raise awareness about dementia (15%); advocate for people living with dementia (4%); volunteer (2%). Six people included in their response more than one thing that they will be doing to make their community more dementia-friendly. Responses to the community board question indicate that the mural encouraged some members of the public to act. Additionally, the artists who created the pieces wanted their work to encourage community members to learn more about dementia and to recognize the importance of living in a supportive and inclusive community, both of which align with the two top categories, indicating that the artwork resulted in the intended outcome. Although we did not assess whether people followed through with their proposed actions, findings reinforce the theme that the mural has planted a seed for change.

**Table 4.** Community boards answers to the question “What will you do to make our community more dementia-friendly?”.

**Support all community members, regardless of their diagnosis** *“Be there more for people who need assistance no matter for what. If seeing a need, not wait to ask but offer help and respect what the person would like, to be helped or not to be helped.”*	19 (32%)
**Seek more dementia education** *“I will commit to learning and understanding those living with dementia and their experiences- ask them in which ways I can help.”*	18 (30%)
**Raise awareness about dementia** *“I will inspire people to learn and ask questions as knowledge & awareness combats stigma.”*	15 (25%)
**Advocate for people living with dementia** *“Continue to advocate for home health services to allow people to remain at home longer. Caregivers such as spouses and/or children need increased support in term of caregiver relief.”*	4 (7%)
**Volunteer** *“Volunteer wherever I can help.”*	2 (3%)
**Nothing**	2 (3%)

Moreover, from the 48 community board responses to the question “*How does it feel to have this mural in our community?*” five categories were identified in the analysis (refer to Table 5): amazing (34%); inspiring (32%); inclusive (22%); source of pride (11%); impartial (1%). Responses to the community board question indicate that the mural elicited positive feelings in most people that contributed to the community boards.

**Table 5.** Community boards answers to the question “How does it feel to have this mural in our community?”.

**Amazing** *“Amazing! A real honour.”*	15 (34%)
**Inspiring** *“It feels ground-breaking and exciting! This is change in the making and I am inspired.”*	14 (32%)
**Inclusive** *“It is nice to see representation of all kinds of people in Haldimand county, including people living with dementia”*	10 (22%)
**Source of pride** *“It feels good to acknowledge that there are people with dementia living in our community. Very proud of it!”*	5 (11%)
**Impartial**	4 (1%)

### Did the mural encourage the public to have conversations about dementia and/or DFCs with their social circle?

From the 34 online-community board responses to the question, *“Have you have conversations about the mural with your friends or family?“*16 (47%) indicated that they have had conversations with their social circle, 6 (18%) indicated that they have not had conversations with their social circle, and 12 (35%) indicated that they had not had conversations with their social circle yet, but they plan on having one. From the 16 people who reported having conversations with their social circles about the mural, five categories of the topics discussed in their conversations were identified in the analysis (refer to Table 6): the mural (38%); dementia (25%), the MICE team (19%); helping others (12%) and research (6%). Refer to Table 7 for a summary of the categories. Similarly, from the 12 people planning to have a conversation with their social circle, three categories of discussion topics were identified in the analysis: dementia awareness and education (67%); how to support persons living with dementia (25%); and the MICE team (8%). Responses to the online-community board questions indicate that the mural encouraged some people to have conversations with their social circle about dementia, and other topics related to the work of the MICE team. These findings further reinforce that the mural has planted a seed for change.

**Table 6.** Online-community board answers to the question “What conversations are you planning on having with your friends/family?”

**Dementia awareness and education** *“Advocating for people living with dementia and talking about what dementia is.”*	8 (67%)
**How to support persons living with dementia** *“The challenges that people with dementia face and how we can help.”*	3 (25%)
**The MICE team** *“Conversation about the MICE team and what they are doing for our community.”*	1 (8%)

**Table 7.** Online-community board answers to the question “What was the conversation with your friends or family about?”.

**The mural** *“We talked about how great it was to have the mural, especially coming from local artists.”*	6 (38%)
**Dementia** *“How the mural inspired me to learn more about dementia.”*	4 (25%)
**The MICE team** *“The MICE team and why what they are trying to do in Haldimand is important.”*	3 (19%)
**Helping others**“*Being more aware of people that need help in the community, step up and help as much as you can.”*	2 (12%)
**Research** *“Progress in research.”*	1 (6%)

### What lessons can be learned from the process undertaken to create this community mural?

Findings from all data sources were used to compile a list of lessons learned through the process of creating this community mural:

1. For a project to be truly inclusive, it is essential to involve persons living with dementia in planning and implementation processes.
2. Having persons living with dementia leading the development and implementation of community projects is a powerful way of combating stigma.
3. A strength of a community mural is that both the process of creating the mural, and the outcome can increase awareness and impact.
4. After the launch of the mural, more follow-up activities would have been helpful to increase awareness and engagement with the mural.
5. A social media campaign could have increased engagement from different age groups.
6. It is important to think about the location of the mural and how people will be interacting with it.
7. It is important to consider which medium will be used to display the artwork. Removable aluminum panels provide the possibility of reinstalling the mural in another location/community.
8. To raise awareness about the mural, leveraging other community projects, such as the local fall fair, may have been helpful.
9. One of the most impactful things about the launch event was having the MICE team sharing their experiences of living with dementia, which provided an opportunity to educate the community about dementia.
10. The MICE festival was intended to bring attention to the mural; however, the mural was a 10-min walk from the festival and not everyone who attended the festival went to see the mural. Thus, a specific event by the mural may have brought more attention to the mural.
11. Embedding an evaluation component helped the MICE team to stay focused on what they wanted to achieve, added credibility to their work, and elements of the evaluation (e.g., community boards) created opportunities to further encourage people to think about how they can help create more inclusive communities.

Lastly, from the interviews and focus group we learned that the project had a positive impact on everyone involved. As described by two MICE team members “*it [this project] brought us back into the community of doers. That on itself was gigantic.”* (05) and “*I’ve been sitting at home for year, doing nothing. This project was amazing to do… I am surprised that it became what it did*” (04). As illustrated in the comments, this project provided MICE team members with an opportunity to be meaningfully engaged in a project that was important to them and has empowered them to continue advocating for inclusive communities. Similarly, interview participants shared that involvement with this project resulted in greater awareness about dementia and a shift in their attitudes. As shared by an interview participant, *“you have kind of a stereotype of what someone living with dementia is like, and my stereotype has probably been more on the side of you know, they become very dependent people that don’t have much independent ability. And just to see how they [MICE team members] deal with their challenges, that brought more awareness to me of the scope of what people with dementia are able to do.”* (08). Thus, findings suggest that an impactful element of dementia awareness raising initiatives is contact between persons living with dementia and members of the public.

## Discussion

The aim of this outcome evaluation was to assess the impact of MICE’s community mural and to capture lessons learned from the process undertaken to create the mural. Findings from this study add to our understanding of the potential impact that community murals could have on social change, particularly on raising dementia awareness and starting conversations about dementia and DFCs. Our findings indicate that the process of creating the mural, and the mural itself, have planted a seed for increased dementia awareness and inclusion of persons living with dementia in Haldimand County.

### Impact of the process undertaken to create the mural

#### Impact on stakeholders

As illustrated in Figure 1, the MICE team engaged with community members several times throughout the project. Through these engagements, the MICE team had the opportunity to raise awareness about dementia. In accord with previous research, which has shown that opportunities for contact with a stigmatized group of people is an effective strategy to reduce stigma (see e.g., Griffiths et al., 2014), stakeholders who participated in the interviews shared that contact with the MICE team increased their awareness about dementia and challenged their misconceptions about the capabilities of persons living with dementia. Despite the benefits of contact between persons living with dementia and community members, in most dementia awareness-raising campaigns, persons living with dementia do not engage with community members (Phillipson et al., 2019). Evidence from this evaluation suggests that direct contact between persons living with dementia and community members is an effective way of challenging misconceptions about dementia and reducing dementia-related stigma, emphasizing the importance of providing opportunities for persons living with dementia to be actively involved in planning and implementation processes of dementia awareness campaigns.

#### Impact on MICE team members

As described by persons living with dementia, empowerment is “a confidence-building process whereby [persons living with dementia] are respected, have a voice and are heard, are involved in making decisions about their lives and have the opportunity to create change through access to appropriate resources” (McConnell et al., 2019, p. 9). This definition of empowerment aligns with what MICE team members shared about the impact that this project had on them personally; leading this project made them feel empowered, further increasing their desire to continue advocating for change in their communities. Similarly, persons living with dementia involved in a project from the United Kingdom called the Dementia Engagement and Empowerment Project (DEEP) have expressed that when they are provided with the appropriate supports to lead and manage initiatives, they feel empowered to continue to advocate and create change in their communities (DEEP, 2019; Litherland & Williamson, 2013). The empowerment of MICE team members illustrates that when marginalized populations are given the resources and opportunities to lead projects aimed at improving the well-being of the populations that they represent, there is significant potential for impact at the personal and community levels.

MICE team member’s comments about the positive impact that the project has had on them personally (e.g., “*it [this project] brought us back into the community of doers. That on itself was gigantic.”* (05)) also support the claim that an asset-based approach creates an opportunity for marginalized individuals to rebuild their confidence and self-esteem and regain motivation for participation in meaningful activities (Rahman & Swaffer, 2018). Importantly, by supporting the engagement of persons living with dementia in community development programs, the autonomy and agency of persons living with dementia and their expertise and contributions are acknowledged and respected.

### The impact of the mural

Through this evaluation, we aimed to evaluate the impact of MICE’s mural on community members by assessing whether the mural captured the interest of community members and inspired them to act; encouraged community members to learn more about the work of the MICE team; and/or encouraged them to have conversations about dementia and/or DFCs with their social circle.

Findings from our observations indicate that the mural captured the attention of some community members driving and/or walking by it. However, lack of media requests and engagement with MICE’s website and social media posts suggests that the mural has not encouraged members of the public to learn more about the work of the MICE team, the mural, or to act by starting conversations in social media. Lack of interest from community members to learn more about the meaning of a mural was also found in an evaluation of a mural in Durban, South Africa (Marschall, 1999); even though community members had been passing by the mural regularly, they had not taken a closer look at it and expressed they were not inclined to learn more about it (Marschall, 1999).

As described by Brennan (2019), the creation of participatory public art promotes the engagement of members from the public through the planning, selection, creation, installation, maintenance and ongoing interaction with the final product. Although the MICE team engaged community stakeholders from the planning through the installation stages, they did not create a plan for engagement following the launch of the mural. Upon reflection, further activities and/or opportunities for members of the public to engage with the themes of the mural could have further spread awareness about dementia and DFCs. A recurrent theme that arose from the interviews and focus group was that a social media strategy to encourage further discussion about what members of the public could do to make their communities more dementia-friendly could have been helpful to increase awareness about the mural. Two principles have been proposed for creating public health initiatives suited to social media: creating engaging and stimulating content that encourages people to share it with their network, and encouraging interaction with the content through message boards and user-generated content (Kilaru et al., 2014). e.g., to encourage people to share a picture of the mural with their social network, the MICE team could have developed a contest inviting members of the public to share a picture of the mural along with what a DFC means to them.

One way in which community members were engaged after the launch of the mural was through the online-community boards. The community boards served two purposes, they provided data for this evaluation as well as an opportunity to stimulate reflection about the themes of the mural (i.e., dementia and DFCs). When prompted with questions related to the themes of the mural, 96% of respondents who participated in the community boards indicated that they intended to do at least one thing to make their community more dementia-friendly (e.g., volunteer). Notably, the themes identified aligned with the artists’ intetions of having their art pieces encourage community members to learn more about dementia and to recognize the importance of inclusive communities. Additionally, 82% of respondents who completed the online-community board indicated that they have had or were planning to have a conversation about the mural with their social circle. These findings suggest that using questions to prompt community members to think about the theme(s) of the mural is one way to support community appreciation of the public art piece and to encourage reflection and further discussion. The community boards are one example of what Brennan (2019) refers to as *participatory collective appreciation* –the use of activities and events to promote reflection, encourage dialogue and appreciation of public artwork.

### Limitations

Although in this evaluation we used numerous data collection methods to understand the impact of MICE’s mural, our data collection methods have limitations. First, a small number of community members participated in the community boards, and we did not follow up on whether participants in fact acted, as they had indicated they would, to make their community more dementia-friendly; therefore, based on the findings it can only be assumed that the mural inspired participants to think about what they could do to make their community more dementia-friendly. Additionally, Google analytics provided us with information about webpage visits; however, we were not able to track whether the mural and/or promotional efforts were the reason that users visited the webpage. Moreover, the MICE team, project coordinator and artists have a vested interest in the success of the project, and they all commented on the positive impact that the mural has had. To prevent their answers from biasing results, we used multiple data sources to support evaluation findings and when applicable, provided corroborating evidence from other data sources to understand the impact of the mural. Furthermore, we cannot ensure that each count from our observations represents a unique individual; some individuals could have interacted with the mural more than once. Therefore, findings from our observations should be interpreted with caution. Lastly, all of our materials were in English which prevented participation from community members who cannot read or write in English. Further research is needed to understand whether public art could be used to raise dementia awareness in ethnically diverse communities.

## Conclusion

Community murals and other forms of public art have been used as a method to raise awareness and encourage discussion about issues impacting the community (Burnham, 2011; Szőke & Parizeau, 2019). However, there is limited information about the impact of public art on social change. The aim of this evaluation was to advance our understanding of the impact of community murals by evaluating the impact of a community mural that was created to raise awareness about dementia and DFCs. Our findings indicate that public art can plant a seed for social change, particularly if the piece is displayed for a prolonged period of time, and if throughout the project there are embedded opportunities for community engagement with educational material about the theme(s) being depicted in the art, and/or direct contact with persons living with the condition about which the art piece is trying to raise awareness. Findings from this evaluation suggest that community murals can be one tool that stakeholders can use to raise awareness about dementia; however, as stated by Kent and Nikitin (2012), “public art projects will be most effective when they are part of a larger, holistic, multidisciplinary approach” (para 6). Thus, to create social change, complementary awareness raising initiatives, such as community education workshops are recommended. These additional activities can encourage further conversations about the art piece and personal action.

## References

[bibr1-14713012251333864] Alzheimer’s Disease International . (2016). Dementia friendly communities: Key principles. https://www.alzint.org/u/dfc-principles.pdf

[bibr2-14713012251333864] Botting T. (2022a). What you need to know about MICE Festival Haldimand. The Haldimand Press. https://www.thespec.com/local-haldimand/news/2022/09/04/what-you-need-to-know-about-mice-festival-haldimand.html

[bibr3-14713012251333864] Botting T. (2022b). Making Haldimand more dementia friendly. The Hamilton spectator. https://www.thespec.com/local-haldimand/news/2022/02/14/making-haldimand-more-dementia-friendly.html

[bibr54-14713012251333864] Brennan J. (2019) (In this issue). Public Art and the Art of Public Participation. National Civic League, 108(3), 34–44. DOI: 10.32543/naticivirevi.108.3.0034.

[bibr4-14713012251333864] Burnham L. (2011). A working guide to the landscape of art for change: Community arts at work across the U.S. Animating Democracy. https://www.americansforthearts.org/sites/default/files/CommunityArtsWorkAcrossLBurnhamTrendPaper1_0.pdf

[bibr5-14713012251333864] Cheung M. Guaring G. Smith N. Craven O. (2021). The impacts of public art on cities, places and people’s lives. The Journal of Arts Management, Law, and Society, 52(1), 37–50. DOI: 10.1080/10632921.2021.1942361.

[bibr6-14713012251333864] Ciesielska M. Boström K. W. (2018). Observation methods. In Qualitative methodologies in organization studies (pp. 33–52). Palgrave Macmillan. DOI: 10.1007/978-3-319-65442-3_2.

[bibr7-14713012251333864] Conrad D. (1995). Community murals as democratic art and education. Journal of Aesthetic Education, 29(1), 98–102. DOI: 10.2307/3333522.

[bibr8-14713012251333864] Cousins B. Whitmore E. (1998). Framing participatory evaluation. New Directions for Evaluation, 1998(80), 5–23. DOI: 10.1002/ev.1114.

[bibr9-14713012251333864] Creswell J. Plano C. (2017). Designing and conducting mixed methods research (3rd ed.). Sage Publications.

[bibr10-14713012251333864] DEEP . (2019). What is deep? As seen through the eyes of London DEEP gathering. https://www.dementiavoices.org.uk/wp-content/uploads/2019/07/What-is-DEEP-A5-Booklet.pdf

[bibr11-14713012251333864] Elo S. Kyngäs H. (2008). The qualitative content analysis process. Journal of Advanced Nursing, 62(1), 107–115. DOI: 10.1111/j.1365-2648.2007.04569.x. 18352969

[bibr12-14713012251333864] Fetters M. D. Curry L. A. Creswell J. W. (2013). Achieving integration in mixed methods designs-principles and practices. Health Services Research, 48(6 Pt 2), 2134–2156. DOI: 10.1111/1475-6773.12117. 24279835 PMC4097839

[bibr13-14713012251333864] Frankish H. Horton R. (2017). Prevention and management of dementia: A priority for public health. Lancet (London, England), 390(10113), 2614–2615. DOI: 10.1016/S0140-6736(17)31756-7. 28735854

[bibr14-14713012251333864] Gove D. Downs M. Vernooij-Dassen M. Small N. (2016). Stigma and GPs’ perceptions of dementia. Aging & Mental Health, 20(4), 391–400. DOI: 10.1080/13607863.2015.1015962. 25765096

[bibr15-14713012251333864] Gressel K. (2012). Public art and the challenge of evaluation. https://createquity.com/2012/01/public-art-and-the-challenge-of-evaluation/

[bibr16-14713012251333864] Griffiths K. M. Carron-Arthur B. Parsons A. Reid R. (2014). Effectiveness of programs for reducing the stigma associated with mental disorders. A meta-analysis of randomized controlled trials. World Psychiatry: Official Journal of the World Psychiatric Association (WPA), 13(2), 161–175. DOI: 10.1002/WPS.20129. 24890069 PMC4102289

[bibr17-14713012251333864] Hall T. Robertson I. (2001). Public art and urban regeneration: Advocacy, claims and critical debates. Landscape Research, 26(1), 5–26. DOI: 10.1080/0142639012002445.

[bibr18-14713012251333864] Ho R. T. H. Potash J. S. Ho A. H. Y. Ho V. F. L. Chen E. Y. H. (2016). Reducing mental illness stigma and fostering empathic citizenship: Community arts collaborative approach. Social Work in Mental Health, 15(4), 469–485. DOI: 10.1080/15332985.2016.1236767.

[bibr19-14713012251333864] Kent F. Nikitin C. (2012). Collaborative, creative placemaking: Good public art depends on good public spaces. https://www.pps.org/article/collaborative-creative-placemaking-good-public-art-depends-on-good-public-spaces

[bibr20-14713012251333864] Kilaru A. S. Asch D. A. Sellers A. Merchant R. M. (2014). Promoting public health through public art in the digital age. American Journal of Public Health, 104(9), 1633–1635. DOI: 10.2105/AJPH.2014.302088. 25033155 PMC4151910

[bibr21-14713012251333864] Litherland R. Williamson T. (2013). DEEP: The engagement, involvement and empowerment of people with dementia in collective influencing. Working with Older People, 17(2), 65–73. DOI: 10.1108/13663661311325481/FULL/PDF.

[bibr22-14713012251333864] Marschall S. (1999). A critical investigation into the impact of community mural art. Transformation, 40, 55–86.

[bibr23-14713012251333864] Matsumoto H. Maeda A. Igarashi A. Weller C. Yamamoto-Mitani N. (2021). Dementia education and training for the general public: A scoping review, Gerontology and Geriatrics Education, 44(2), 154–184. DOI: 10.1080/02701960.2021.1999938. 34791985

[bibr24-14713012251333864] McConnell T. Sturm T. Best P. Stevenson M. McCorry N. Donnelly M. Taylor B. J. (2019). Co-producing a shared understanding and definition of empowerment with people with dementia. Research Involvement and Engagement, 5(1), 1–11. DOI: 10.1186/S40900-019-0154-2/TABLES/4. 31205750 PMC6558688

[bibr25-14713012251333864] McEwan C. Szablewska L. Lewis K. V. Nabulime L. M. (2022). Public-making in a pandemic: The role of street art in East African countries. Political Geography, 98, Article 102692. DOI: 10.1016/J.POLGEO.2022.102692. PMC9180876 35702711

[bibr26-14713012251333864] Milne C. Pojani D. (2022). Public art in cities: What makes it engaging and interactive? Journal of Urban Design, 28(3), 1–20. DOI: 10.1080/13574809.2022.2121272.

[bibr27-14713012251333864] Mohatt N. V. Singer J. B. Evans A. C. Matlin S. L. Golden J. Harris C. Burns J. Siciliano C. Kiernan G. Pelleritti M. Tebes J. K. (2013). A community’s response to suicide through public art: Stakeholder perspectives from the finding the light within project. American Journal of Community Psychology, 52(1–2), 197–209. DOI: 10.1007/S10464-013-9581-7. 23743604 PMC3865777

[bibr28-14713012251333864] Nguyen T. Li X. (2020). Understanding public-stigma and self-stigma in the context of dementia: A systematic review of the global literature. Dementia, 19(2), 148–181. DOI: 10.1177/1471301218800122. 31920117

[bibr29-14713012251333864] Parker M. Barlow S. Hoe J. Aitken L. (2020). Persistent barriers and facilitators to seeking help for a dementia diagnosis: A systematic review of 30 years of the perspectives of carers and people with dementia. International Psychogeriatrics, 32(5), 611–634. DOI: 10.1017/S1041610219002229. 32024558

[bibr30-14713012251333864] Phillipson L. Hall D. Cridland E. Fleming R. Brennan-Horley C. Guggisberg N. Frost D. Hasan H. (2019). Involvement of people with dementia in raising awareness and changing attitudes in a dementia friendly community pilot project. Dementia, 18(8), 2679–2694. DOI: 10.1177/1471301218754455. 29363336

[bibr31-14713012251333864] Rahman S. Swaffer K. (2018). Assets-based approaches and dementia-friendly communities. Dementia, 17(2)131–137. DOI: 10.1177/1471301217751533. 29299934

[bibr32-14713012251333864] Renzella M. (2022). DICE mural contest winners announced. The Haldimand Press. https://haldimandpress.com/dice-mural-contest-winners-announced/

[bibr33-14713012251333864] Schrader T. (2017). The colors of loisaida: Embedding murals in community activism. Journal of Urban History, 44(3), 519–532. DOI: 10.1177/0096144217699173.

[bibr34-14713012251333864] SocioCultural Research Consultants LLC . (2019). Dedoose Version 8.3.35, web application for managing, analyzing, and presenting qualitative and mixed method research data. https://www.dedoose.com/

[bibr35-14713012251333864] Statistics Canada . (2021). Profile table, census profile, 2021 census of population - Haldimand county, city (CY) [census subdivision], Ontario. https://www12.statcan.gc.ca/census-recensement/2021/dp-pd/prof/details/page.cfm?DGUIDlist=2021A00053528018&GENDERlist=1&HEADERlist=0&Lang=E&STATISTIClist=1&SearchText=Haldimand+County

[bibr36-14713012251333864] Szőke T. Parizeau K. (2019). Community-based public art and gentrification in the downtown eastside of vancouver. GeoHumanities, 5(1), 157–177. DOI: 10.1080/2373566X.2018.1543554.

[bibr37-14713012251333864] van Wijngaarden E. Alma M. The A. M. (2019). The eyes of others’ are what really matters: The experience of living with dementia from an insider perspective. PLOS ONE, 14(4), Article e0214724. DOI: 10.1371/JOURNAL.PONE.0214724. 30943277 PMC6447241

[bibr38-14713012251333864] World Health Organization . (2012). Dementia a public health priority. World Health Organization.

[bibr39-14713012251333864] World Health Organization . (2021a). Dementia. https://www.who.int/news-room/fact-sheets/detail/dementia

[bibr40-14713012251333864] World Health Organization . (2021b). Towards a dementia inclusive society. https://www.who.int/publications/i/item/9789240031531

